# Comparative Study on the Influence of Noble Metal Nanoparticles (Ag, Au, Pd) on the Photocatalytic Activity of ZnO NPs Embedded in Renewable Castor Oil Polymer Matrices

**DOI:** 10.3390/ma13163468

**Published:** 2020-08-06

**Authors:** Andreea L. Chibac-Scutaru, Viorica Podasca, Daniel Timpu, Violeta Melinte

**Affiliations:** Photochemistry and Polyaddition Department, Petru Poni Institute of Macromolecular Chemistry, 41 A, Grigore Ghica Voda Alley, 700487 Iasi, Romania; andreea.chibac@icmpp.ro (A.L.C.-S.); podasca.viorica@icmpp.ro (V.P.); dtimpu@icmpp.ro (D.T.)

**Keywords:** nanoparticle, castor oil, photopolymerization, nanocomposite, photocatalysis

## Abstract

Hybrid polymeric materials, due to the unique combination of properties that can be obtained by the convenient variation of organic and inorganic components, represent an attractive alternative for many applications, especially photocatalysis. Herein, we report the preparation of nanocomposite films containing functionalized ZnO nanoparticles, as well as in situ photogenerated noble metal nanoparticles (Ag, Au, Pd), for the achieving of materials with enhanced photocatalytic activity under visible light. The flexible free-standing nanocomposite films were synthesized by photopolymerization of a monomer mixture (silane castor oil urethane dimethacrylate and polypropylene oxide urethane dimethacrylate) in the presence of a Irgacure 819 photoinitiator. The efficiency of ZnO NPs functionalization was established by Fourier transform infrared spectroscopy (FTIR) and thermogravimetric analysis, while the polymer composites were characterized by UV-Vis spectroscopy, X-ray diffraction, transmission electron microscopy and scanning electron microscopy to evidence the formation, size and distribution of the nanoparticles inside the photocrosslinked matrix. To establish the photocatalytic capacity of nanocomposite films, the decomposition of various pollutants (methyl orange, phenol, metronidazole) was monitored under visible light irradiation, the best results being obtained for Au/ZnO film. Also, the advantage of immobilizing the catalysts in a polymeric support and its recycling ability without a significant decrease in photocatalytic efficiency was analysed.

## 1. Introduction

The continuous development of industry and society leads to the disposal of an increasing amount of polluting waste, especially for water pollution, which has become a serious environmental problem since many industrial effluents containing organic and inorganic impurities are constantly discharged into the water flow. Among the most harmful are considered recalcitrant organic pollutants (dyes, antibiotics and pesticides) with a noxious impacts on both humans and the environment [1]. An intensively investigated method for water treatment is photocatalysis, which has been recognized as a convenient, green and inexpensive technology for the complete decomposition of organic pollutants into H_2_O and CO_2_ [2,3,4]. Up to now, numerous semiconductors (TiO_2_, ZnO, CdS, ZnS, CdTe, *α*-Fe_2_O_3_, BiOI or SrTiO_3_) were applied as photocatalysts [5,6,7,8,9,10]. Among them, ZnO has become known as an efficient and promising candidate in pollutants removal due to its unique characteristics, such as direct and wide band gap (3.37 eV), good photocatalytic property, large area-to-volume ratio, environmentally friendly nature and low cost [11]. However, the wide band gap of ZnO nanostructures limits their use in the visible-light region of the solar spectrum, reason for that many approaches have been made to narrow the band gap and to shift the absorption of ZnO photocatalyst to visible-light. A viable alternative to inhibit charge recombination and to improve the visible light absorption of ZnO is represented by the doping or coupling with noble metal nanoparticles (Au, Ag, Pt, Pd) [12,13,14].

The use of inorganic nanoparticles as photocatalysts is often accompanied by some disadvantages, namely their agglomeration that caused a decrease of the surface area and implicitly of the photocatalytic potential and their recovery and reusing processes that are difficult and costly [15,16]. An efficient pathway to overcome these problems is to immobilize photocatalysts on various supports, such as glass, carbonaceous substances, zeolites, ceramics, cellulosic materials or polymers [17]. Polymeric materials were frequently used as templates to incorporate the inorganic nanoparticles due to their excellent properties such as high processability, film forming abilities, architectural diversity and low-cost [18,19,20,21]. The use of polymeric materials derived from renewable resources, such as vegetable oils [22,23] or cellulose [24,25] in the immobilization of inorganic nanoparticles could enhance the versatility of the resulting hybrid coatings. Furthermore, the preparation of hybrid materials through photopolymerization benefits from the advantages offered by photocuring technology (very simple, low energy consumption and process costs, environmentally friendly with almost no release of volatile organic compounds) that allow the achieving of high quality tailorable networks [26]. The simultaneous photogeneration of noble metal nanoparticles (Ag, Au, Pd) in tandem with the photopolymerization process lead through a simple and efficient procedure to a homogeneous distribution of metal nanoparticles (NPs) inside the crosslinked nanocomposites [27,28,29].

Based on the above considerations, in this study we present the design, preparation and the comparative photocatalytic activity of some polymeric hybrid materials based on ZnO NPs and in situ synthesized Ag, Au and Pd metal nanoparticles. The organic template was achieved upon the photopolymerization of two urethane dimethacrylate monomers (one derived from bio-based castor oil with silane sequences and other from polypropylene oxide). ZnO NPs were initially functionalized with 3-(trimethoxysilyl)propyl methacrylate to prevent their agglomeration and diffusion from the polymeric matrix, while in order to shift the catalytic activity to visible light, Ag, Au and Pd NPs were in situ photogenerated during the photopolymerization process.

## 2. Materials and Methods

### 2.1. Materials

3-(Trimethoxysilyl)propyl methacrylate, castor oil (CO), 2-isocyanatoethyl methacrylate, 3-(triethoxysilyl)propyl isocyanate, poly(propylene glycol) M_w_ = 1000, trimethyl-1,6-diisocyanatohexane, mixture of 2,2,4- and 2,4,4-isomers (TMDI), 2-hydroxyethyl methacrylate, anhydrous tetrahydrofuran (THF), toluene, dibutyltin dilaurate, AgNO_3_, gold chloride trihydrate (HAuCl_4_·3H_2_O), Pd(NO_3_)_2_·2H_2_O and Irgacure 819 were purchased from Sigma Aldrich Chemical Co (Taufkirchen, Germany) and used without further purification. The synthesis protocol for the preparation of ZnO nanoparticles was reported into a previous publication [30].

### 2.2. Functionalization of ZnO Nanoparticles

The functionalization of ZnO nanoparticles through the sol-gel method was performed by a published literature method [31]. Thus, 3 g ZnO nanoparticles were dispersed by ultrasonication into 100 mL anhydrous toluene and then an excess of 3-(trimethoxysilyl)propyl methacrylate was added dropwise under vigorous stirring for one hour. Next, the mixture was kept under stirring, under argon atmosphere and at room temperature for 24 h. The functionalized nanoparticles (Sil-ZnO) were separated by centrifugation, washed twice with toluene and methylene chloride and dried under vacuum for 12 h, at 35 °C.

### 2.3. Synthesis of Photopolymerizable Castor Oil Urethane Dimethacrylate with Silane Sequences (CO-UDMA-Si)

**Figure 1:**
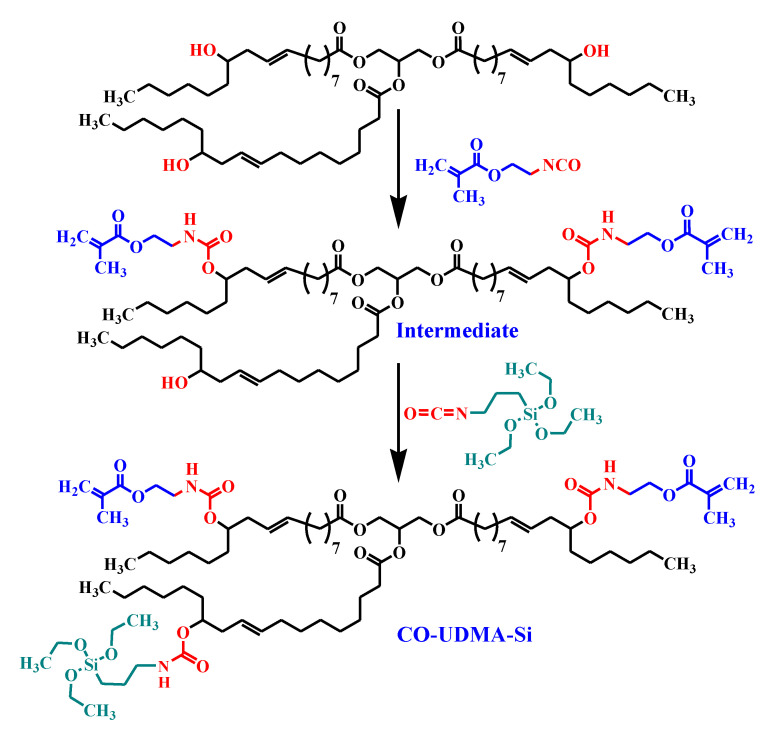
Schematic diagram of Castor Oil Urethane Dimethacrylate with Silane Sequences (CO-UDMA-Si) monomer preparation.

For the synthesis of CO-UDMA-Si monomer (Figure 1), 10 g (10.7 mmol) CO were degassed under vacuum for 2 h.

Further, 3.1 mL (21.4 mmol) 2-isocyanatoethyl methacrylate dissolved in 20 mL THF was dropwise added and the mixture was stirred at 40 °C for 12 h in the presence of catalytic amount of dibutyltin dilaurate. The progress of the reaction was controlled by Fourier transform infrared (FTIR) spectroscopy following the absorption of the isocyanate stretching band at 2260 cm^−1^ up to its disappearance from the spectrum. In the second step, 10 g of intermediate castor oil urethane dimethacrylate were dissolved in anhydrous THF and 2.1 mL (8.05 mmol) 3-(triethoxysilyl)propyl isocyanate were added, the reaction mixture being kept under stirring at 40 °C for 12 h, following the same protocol as for the intermediate preparation. After the solvent evaporation, the urethane dimethacrylate monomer CO-UDMA-Si was achieved as a colourless viscous liquid.

**CO-UDMA-Si**. ^1^H NMR (CDCl_3_, *δ* ppm): 6.06 (2H, CH_2_=C in trans position relative to CH_3_ unit); 5.52 (2H, CH_2_=C in cis position relative to CH_3_ group); 5.27–5.37 (6H, –CH=CH– from CO); 4.97 (1H, –CH–OCO–CH_2_); 4.15 (8H, –CH_2_–O–CO–); 4.07 (3H, –CH–OCO–NH–); 3.75 (6H, CH_3_–CH_2_–O–Si); 3.42 and 3.10 (6H, CH_2_–NH–COO); 3.10 (4H, –CH=CH–CH_2_–CO–); 2.25 (6H, –CH–CH_2_–CH=CH–); 1.88 (6H, CH_3_ linked to double bond); 1.5–0.88 (aliphatic protons from CO); 1.23 (9H, CH_3_–CH_2_–O–Si); 0.56 (CH_2_–Si). FTIR (KBr, cm^−1^): 3390 (NH); 2855–2928 (C–H); 1722 (C=O); 1638 and 815 (CH_2_=C); 1534 (amide II); 1250 and 1167 (C–O–C); 1039 (Si–O).

### 2.4. Synthesis of Photopolymerizable Monomer PPO-UDMA

For the preparation of polypropylene oxide urethane dimethacrylate (PPO-UDMA) monomer, 10 g (10 mmol) poly(propylene glycol) were degassed under vacuum, at 90 °C for 2 h. Then, the temperature was reduced to 65 °C and 4.2 mL (20 mmol) TMDI were dropwise added. The reaction was continued for 6 h, at the same temperature, in nitrogen atmosphere under stirring. Further, the temperature was decreased to 38 °C and 2.45 mL (20 mmol) 2-hydroxyethyl methacrylate was added together with 50 mL anhydrous THF. The mixture was kept under stirring at 38 °C for 24 h, in the presence of catalytic amounts of dibutyltin dilaurate and the completion of the reaction was verified by FTIR spectroscopy. The product was achieved by the solvent removal on a rotary evaporator as a viscous light yellow liquid.

**PPO-UDMA**: ^1^H NMR (CDCl_3_, *δ* ppm): 6.15 (2H, CH_2_=C in trans position relative to CH_3_ unit from HEMA); 5.56 (2H, CH_2_=C in cis position relative to CH_3_ unit from HEMA); 4.43–4.32 (8H, NH–CO–O–CH_2_–CH_2_–O–CO–); 3.64–3.30 (33H, –O–CH(CH_3_)–CH_2_–O–); 3.07 (m, 8H, O–CO–NH–CH_2_); 1.93 (6H, –CH_3_ linked to double bond); 1.51–1.07 (10H, methine and methylene protons from TMDI); 1.22 (36H, –CH_3_ protons from PPO); 0.92–0.85 (18H, –CH_3_ protons from TMDI). FTIR (KBr, cm^−1^): 3346 (NH); 2872–2970 (CH); 1721 (C=O); 1638 and 816 (CH_2_=C); 1534 (amide II); 1248 and 1107 (C–O–C).

The prepared dimethacrylates are soluble in volatile organic solvents (tetrahydrofuran, chloroform, methylene chloride, ethanol or acetone) and display very good thin film-forming abilities after the light curing process.

### 2.5. Preparation of Hybrid Composites

For the achieving of hybrid composites, 70:30 wt.% mixtures of photopolymerizable monomers CO-UDMA-Si and PPO-UDMA together with Irgacure 819 (1 wt.%) photoinitiator were prepared. Additionally, in the experimental formulations, functionalized ZnO nanoparticles and different amounts of metal salts (AgNO_3_, HAuCl_4_, Pd(NO_3_)_2_) were included, the mass percentages of the proposed photopolymerizable blends being listed in Table 1.

The quantity of metal precursors was calculated so that in the final photocrosslinked films, the theoretical amount of noble metal nanoparticles to be about 1 wt.%. The compositions were homogenized by adding few drops of acetone to the formulations and were further coated on Teflon plates and photopolymerized under UV irradiation for a period of 10 min at a light intensity of 30 mW cm^−2^. The in situ formation of metal nanoparticle was readily observed by the modification of the films colour—the one with silver NPs is dark brown, Au NPs give a purple colouration while Pd NPs colour the film in black.

### 2.6. Characterization

The structure of urethane dimethacrylate monomers was confirmed by proton nuclear magnetic resonance (^1^H NMR) spectroscopy using a Bruker Avance DRX 400 spectrometer and CDCl_3_ as deuterated solvent (Karlsruhe, Germany). Fourier transform infrared (FTIR) spectra were recorded on a Bruker Vertex 70 FT-IR spectrometer (Karlsruhe, Germany). Thermogravimetric analysis (TGA) was performed on a STA 449 F1 Jupiter apparatus (Netzsch-Germany) in the temperature range of 25–700 °C under a dry nitrogen atmosphere, at a heating rate of 10 °C min^−1^. The amount of grafted silane derivative was calculated from the difference in weight loss between initial ZnO NPs and the modified ones. The X-ray diffraction (XRD) patterns were recorded on a D8 ADVANCE Bruker X-ray Diffractometer (Karlsruhe, Germany) with a Cu Kα (*λ* = 1.54 Å) source in the 2*θ* range from 4.0° to 80.0° in steps of 0.02°. The measurements were performed on powder (Sil-ZnO) or photopolymerized solid films (M1, M1-Ag, M1-Au, M1-Pd).

Transmission electron microscopy (TEM) analyses were carried out using a HITACHI T7700 microscope (Tokyo, Japan) operated at 120 kV in high-resolution mode (samples M1, M1-Ag and M1-Au) and a FEI Titan CT microscope (Hillsboro, Oregon, USA) operating at 300 kV (sample M1-Pd). The morphology of photopolymerized samples was investigated using an ultra-high-resolution field-emission Scanning Electron Microscope VeriosG4 UC (Thermo Fisher Scientific, Brno, Czech Republic) equipped with an energy dispersive spectrometer (EDS, EDAX Octane Elite, City). The samples were fractured in liquid nitrogen and the film cross-sections were examined at an accelerating voltage of 5kV using an Everhart-Thornley SE detector. To achieve high-quality information from the SEM images, the samples were sputter coated with a thin layer of platinum.

### 2.7. Photocatalytic Activity Measurements

First, the photocatalytic activity of the synthesized hybrid films with Sil-ZnO NPs (M1, M1-Ag, M1-Au and M1-Pd) was evaluated as previously described [20]—the photodegradation reaction of methyl orange, in aqueous solutions (100 mL MO, C = 5 × 10^−5^ M) in ambient conditions under visible irradiation and in the presence of each composite film (1 g) was followed. Prior to UV irradiation, the nanocomposite and pollutant mixtures were stirred in under ambient conditions for 20 min each sample. Further, the mixtures were irradiated with a visible light source up to 300 min (Xe lamp *λ* = 400–800 nm, Hamamatsu Lightningcure Type LC8, Model L9588, Iwata City, Shizuoka Pref., Japan) under constant stirring. The progress of the photodegradation reaction was evaluated on a UV-vis spectrophotometer (Perkin Elmer Lambda 2, Wellesley, MA, USA), when 1.5 mL of the irradiated solution was collected and analysed at given time intervals. The reusability of M1-Au catalyst was tested by using the same film in five successive cycles for MO photodegradation. After these repetitive cycles, the M1-Au film was kept in water for 24 h and then another 24 h under normal environmental conditions (air, ambient light and room temperature) and used again in the sixth photodegradation cycle of MO.

Secondly, M1-Au film was tested as catalyst in the photodegradation of metronidazole and phenol aqueous solutions, the experimental procedure being the same as in the case of MO. Also, control experiments were performed and the pollutants solutions (methyl orange, metronidazole, phenol) without hybrid films were irradiated with visible light for 300 min and after that analysed through UV-vis spectroscopy.

## 3. Results and Discussion

### 3.1. Functionalization and Characterization of ZnO Nanoparticles

**Figure 2:**
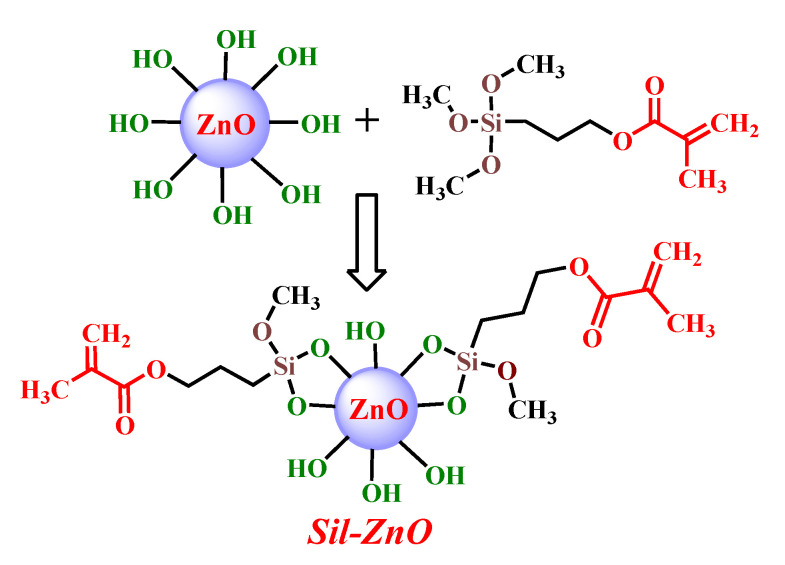
Schematic representation of the functionalization of ZnO nanoparticles.

The chemical functionalization of ZnO nanoparticles is possible due to the presence on their surface of a variable number of hydroxyl groups, capable of interacting under predetermined conditions with various low molecular weight compounds, the alkoxysilane derivatives being preferred [32,33,34]. Thus, in this study, ZnO nanoparticles were functionalized with 3-(trimethoxysilyl)propyl methacrylate (Figure 2), envisaging that silane units will react with the OH groups of ZnO NPs, while the methacrylate sequences will be available to participate in the further photopolymerization reaction along with the selected urethane dimethacrylate monomers CO-UDMA-Si and PPO-UDMA.

**Figure 3:**
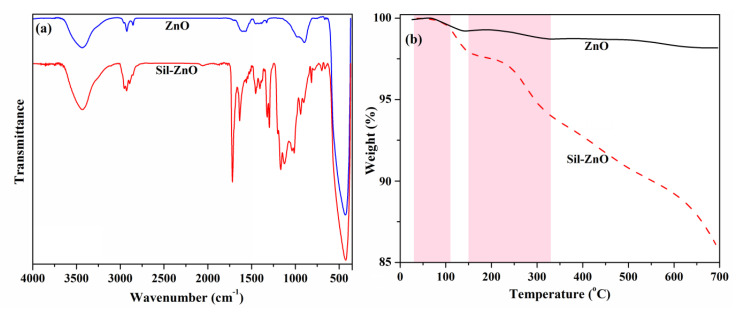
Fourier transform infrared (FTIR) spectra (**a**) and thermogravimetric analysis (TGA)thermograms (**b**) of ZnO and Sil-ZnO nanoparticles.

To highlight the differences appeared after functionalization, both the modified and the initial ZnO NPs were characterized by FTIR spectroscopy (Figure 3a).

In the FTIR absorption spectrum of the pristine ZnO, the intense band with the maximum at 430 cm^−1^ is due to the stretching frequency of Zn–O bonds, while the broad peak at around 3435 cm^−1^ is given by the stretching band of O–H groups on the surface of ZnO NPs [35]. After functionalization, besides the wide absorption band characteristic to Zn–O stretching vibrations at 430 cm^−1^, additional peaks appeared in the spectrum, confirming the functionalization process. Thus, it can be observed the absorption bands given by the Si–O–Si asymmetric vibration at 1040 cm^−1^, the Zn–O–Si stretching vibration located at 939 cm^−1^ and the absorption peaks observed in the 2854–2955 cm^−1^ range are characteristic to the C–H stretching vibrations in CH_2_ and CH_3_ units. Also, the new and intense absorption band located at 1719 cm^−1^ corresponds to C=O stretching, while the absorption bands at 1638 and 816 cm^−1^ are attributed to the methacrylate double bond vibrations. The board absorption peak centred at 3435 cm^−1^ was attributed to the stretching vibration of hydroxyl groups formed during the sol-gel process [15].

The amount of organo-silane derivative bound on the surface of ZnO NPs could be evaluated by thermogravimetric analysis, by measuring the difference of the remaining weight between the initial sample and the silane-modified nanoparticles. As can be observed in Figure 3b, in the first decomposition step (from 30 to 110 °C) the release of the physically entrapped water (which represents approximately 0.5 wt.% for both samples) takes place. Subsequently, ZnO nanoparticles probably lose the chemically absorbed water or some organic fragments that were not completely removed during the calcination process [36], the total weight loss up to 700 °C being of 1.8 wt.%. In the case of Sil-ZnO sample, the weight loss in the 150–330 °C was attributed to the combustion of the organic fragments of the silane modifier, followed by a gradual decrease of sample weight up to 700 °C. The total mass loss determined for Sil-ZnO sample was 13.9 wt.% and the weight loss attributed to silane moieties was about 12.1 wt.%, which represents about 55 mmol 3-(trimethoxysilyl)propyl methacrylate attached at 100 g ZnO NPs.

### 3.2. Preparation and Characterization of Hybrid Composites

**Figure 4:**
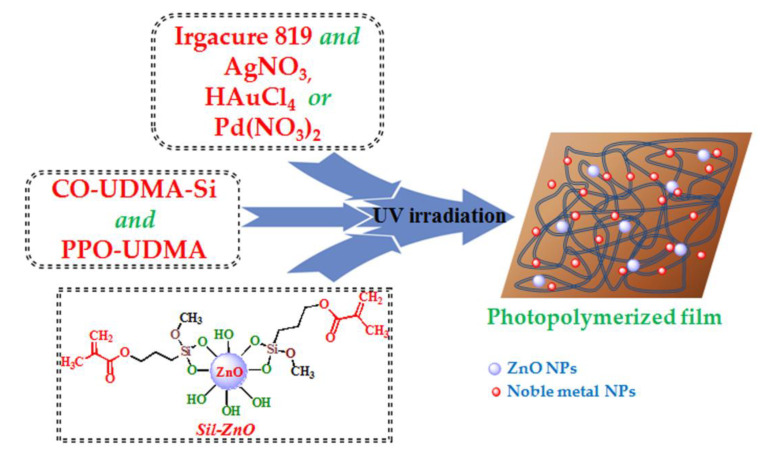
Schematic illustration of the concomitant photopolymerization of methacrylate monomers and in situ photoreduction of metallic salts under UV irradiation with the formation of hybrid polymer networks.

A convenient and straightforward approach frequently used to insure a facile handling, employment and recovery of the inorganic nanoparticles consists in their homogeneous dispersion into polymer matrices, which besides reducing their agglomeration tendency also enhance their chemical stability [18]. Hence, ZnO NPs previously functionalized with photopolymerizable sequences were incorporated in urethane dimethacrylate mixture (gravimetric ratio given in Table 1) and were photopolymerized by UV light irradiation for 600 s in the presence of Irgacure 819 as photoinitiator. Additionally, noble metal nanoparticles (Ag, Au, Pd) were in situ photogenerated in the hybrid matrices concomitant with the polymerization process by adding the corresponding noble metal salts (AgNO_3_, HAuCl_4_ or Pd(NO_3_)_2_), which determines the formation of coatings with a homogeneous distribution of nanoparticles (Figure 4).

In these conditions, the photocrosslinking of the methacrylic matrix took place simultaneously with the photochemical reduction of Ag^+^, Au^3+^ or Pd^2+^ to metallic Ag^0^, Au^0^ or Pd^0^ nanoparticles, processes triggered by the photolysis of Irgacure 819 initiator under UV irradiation [22,29,37]. In the achieving of hybrid nanocomposites, an important role is granted to the polymer matrix composed mainly of bio-based castor oil derivative (CO-UDMA-Si) which together with the polypropylene urethane dimethacrylate (PPO-UDMA) allows the formation of homogeneous, flexible and transparent films in which the inorganic nanoparticles can be easily incorporated.

**Figure 5:**
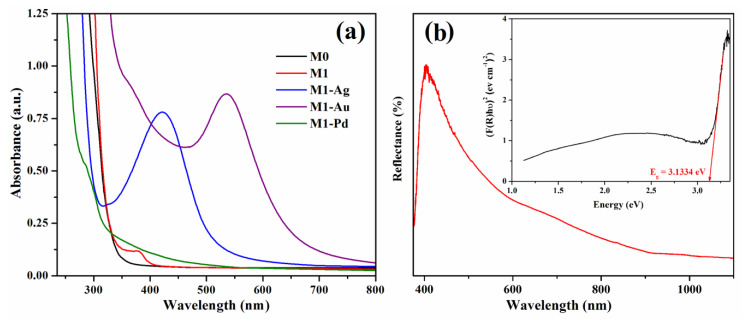
UV-vis absorption spectra for M0, M1, M1-Ag, M1-Au and M1-Pd samples (**a**) and reflectance spectrum of M1 film (**b**). Inset of (**b**) representation of Kubelka–Munk function for M1 sample.

The in situ photogeneration of noble metal nanoparticles (Ag, Au, Pd) in the photocrosslinked matrices was firstly evidenced by the formation in the UV absorption spectra of specific absorption bands given by the surface plasmon resonance effect characteristic to these nanoparticles (Figure 5a).

It can be observed that the pristine M0 film is transparent and the incorporation of Sil-ZnO nanoparticles in M1 sample determines the appearance of a small shoulder at 380 nm. The photogeneration of Ag nanoparticles triggers the formation of the absorption band at *λ_max_* = 421 nm, while gold nanoparticles give the characteristic plasmon at *λ_max_* = 535 nm. In the UV-vis spectrum of M1-Pd film there is no significant increase in absorption in the visible region due to the surface plasmonic absorption of palladium particles, the finding being attributed, according to literature data [38], to the fact that Pd particles smaller than 10 nm are able to absorb only in the UV region of the spectrum.

In order to estimate the band gap (*E_g_)* values of our hybrid composites, the diffuse reflectance UV-vis spectra were also measured and analysed. The band gap energy of the catalyst can be determined from the optical reflectance data by the Kubelka–Munk function (1) by plotting (F(R)*hυ*)^2^ versus photon energy (eV) [39,40,41], Equation (1):

F (R) = (1 − R)^2^/2R, (1)

where R is the observed reflectance in UV-vis spectra, *hυ* is the absorption energy (h—Planck’s constant, *υ*—the frequency of light).

However, this method has a limitation and cannot be accurately applied to dark films when light absorption reaches a high level [42]. Our hybrid films that also contain noble metal nanoparticles, in addition to ZnO NPs, have very dark colours, namely—M1-Ag is dark brown, M1-Au is dark purple and M1-Pd is black. Therefore, the band gap can be accurately determined from the reflectance spectrum only for M1 film containing Sil-ZnO NPs (Figure 5b), the achieved value being E_g_ = 3.133 eV (Figure 5b inset), which indicates UV light absorption capacity. The red shift of the absorption bands of the hybrid composites containing additionally noble metal nanoparticles (Figure 5a) indicates that the band gap energies of M1-Ag, M1-Au and M1-Pd films are lower than that of M1 film [43], demonstrating that they have visible light absorption capacity.

**Figure 6:**
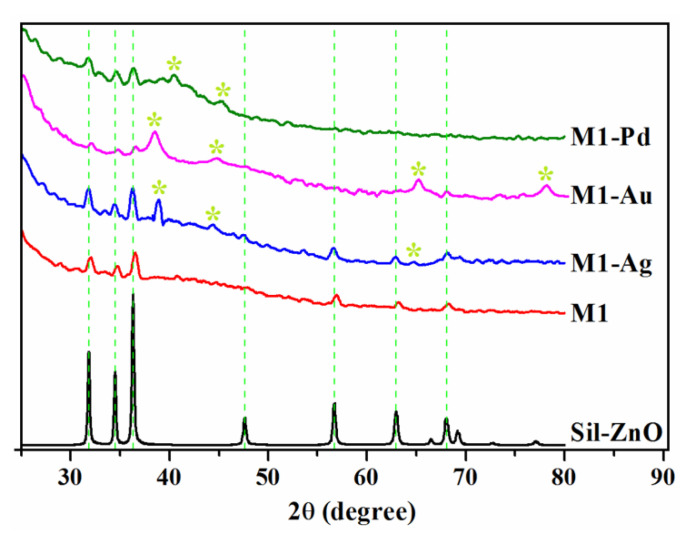
X-ray diffraction (XRD) patterns of Sil-ZnO nanoparticles and of M1, M1-Ag, M1-Au and M1-Pd hybrid films (* indicates the diffraction peaks given by noble metal nanoparticles Ag, Au and Pd, respectively).

Wide angle XRD patterns for the functionalized ZnO NPs (Sil-ZnO) and for the hybrid polymeric films containing Sil-ZnO and noble metal nanoparticles (M1, M1-Ag, M1-Au and M1-Pd) were illustrated in Figure 6.

According to the diffraction pattern of Sil-ZnO powder sample, the diffraction peaks at 31.8, 34.4, 36.3, 47.6, 56.6, 62.9, 69.1 and 77.0 correspond to (100), (002), (101), (102), (110), (103), (200), (201) and (202) planes of the hexagonal ZnO phase [44].

The average crystallite size of ZnO nanoparticles was determined by applying the Debye-Scherrer Equation (2) [19]:

D = Kλ/β cosθ,(2)

where D is the average crystallite size, *λ* is the wavelength of the X-ray radiation (*λ* = 0.154056 nm for Cu Kα radiation), K is the Scherrer constant (K = 0.89), *β* is the full width at half-maximum (FWHM) of the diffraction peak corresponding to (101) plane and *θ* is the diffraction angle. Thus, the average crystallite size calculated for Sil-ZnO NPs was found to be 25 nm.

In the X-ray diffraction patterns of hybrid polymeric films containing Sil-ZnO and noble metal NPs, it can be observed that the diffraction peaks are weaker probably due to the low nanoparticle loading (around 1 wt.%) as well as to the presence of the amorphous organic matrix that interferes with the nanoparticle peaks and give a broad peak around 2 theta value of 20° (not illustrated in Figure 6). Besides the peaks corresponding to ZnO, in the diffraction patterns of M1-Ag, M1-Au and M1-Pd films are noticeable some peaks characteristic to monometallic Ag (39.1°, 44.3°), Au (38.4°, 44.5°, 64.8°) Pd (40.2°, 45.3°) NPs.

**Figure 7:**
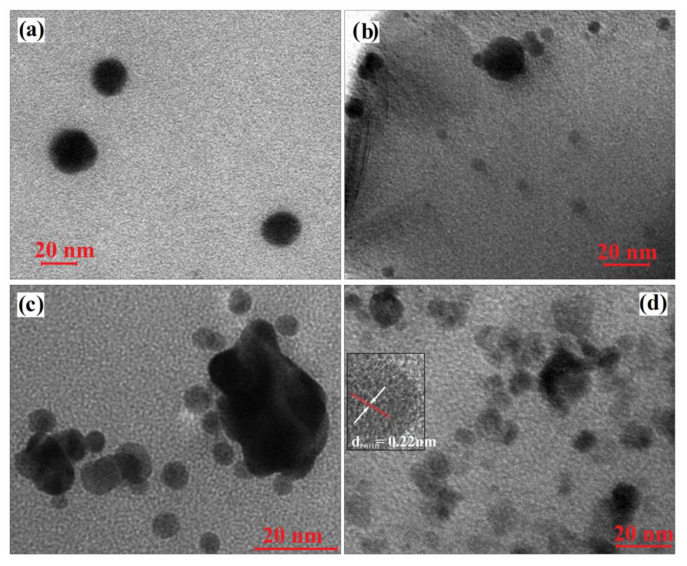
Transmission electron microscopy (TEM) images of M1 (**a**), M1-Ag (**b**), M1-Au (**c**) and M1-Pd (**d**) nanocomposites.

A confirmation of the UV and XRD data, as well as a more accurate proof about the dimensional distribution and the shape of noble metal nanoparticles photogenerated in polymer matrices were obtained by transmission electron microscopy (TEM). Thus, the investigation of M1 composite film (Figure 7a) shows the presence of Sil-ZnO nanoparticles with dimensions around 20 nm (outcome in agreement with XRD data where the crystallites sizes are calculated to be around 25 nm) that seems to be well dispersed in the organic template.

According to this observation, in all TEM micrographs (Figure 7b–d), the larger nanoparticles were assumed to be ZnO NPs, although the crowding of noble metal NPs in the form of clusters provoked by the crosslinking of polymer networks is not excluded.

Regarding the noble metals nanoparticles in situ photogenerated, it can be observed that in Figure 7b, silver NPs are spherical in shape with dimensions below 8 nm, while gold NPs are also spherical and their dimensions are between 5–8 nm (Figure 7c). The TEM image recorded for M1-Pd film (Figure 7d), shows the presence of numerous small nanoparticles with dimensions between 5–10 nm which are assumed to be Pd nanoparticles. Taking into account that is the first time we in situ synthesize Pd NPs, the presence of lattice fringes was highlighted by high-resolution transmission electron microscopy (HRTEM) in which the inter-planar spacing of 0.22 nm corresponds to the (111) planes of face centred cubic Pd NPs [38]. Also, from the TEM images it can be noticed that some of the metal nanoparticles are formed around or in contact with ZnO NPs, which will influence the subsequent catalytic properties of the samples.

**Figure 8:**
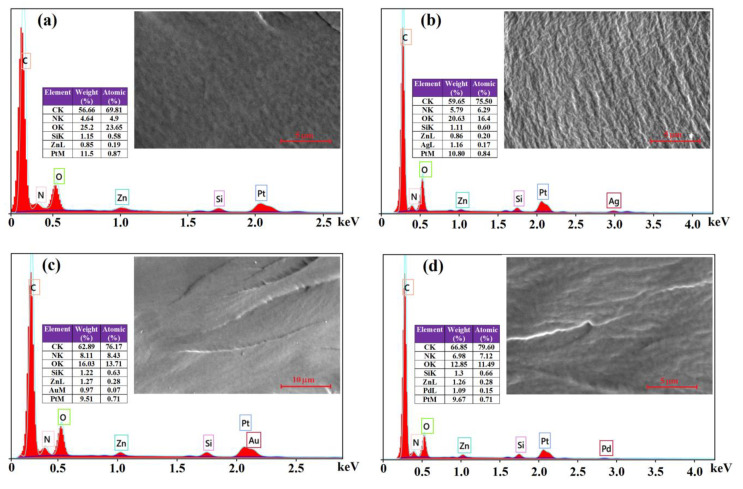
Energy-dispersive X-ray spectroscopy (EDX)spectra and scanning electron microscopy (SEM)micrographs of fractured cross-sections of composite films M1 (**a**), M1-Ag (**b**), M1-Au (**c**) and M1-Pd (**d**).

The chemical composition (qualitative and quantitative) of the photopolymerized nanocomposites incorporating functionalized ZnO and Ag, Au or Pd NPs was investigated by energy-dispersive X-ray spectroscopy (EDX). The EDX patterns illustrated in Figure 8, confirmed the presence of inorganic nanoparticles by Zn, Ag, Au and Pd signals, while C, O and N peaks are characteristic to the organic matrix.

The intense Pt signal in the EDX spectrum is due to the sputter coating of the samples with platinum. The weight percentage of noble metal nanoparticles in the composites is around 1 wt.%, in agreement with the calculated amount that should result from the initial metal salts (AgNO_3_, HAuCl_4_ or Pd(NO_3_)_2_). In addition, the SEM images of fractured composite films (insets of the EDX spectra in Figure 7) display relatively uniform and regular surfaces, suggesting a homogeneous distribution of inorganic components within the polymer matrix.

### 3.3. Photocatalytic Properties

The prepared nanocomposites have potential to be used as photocatalysts with good performance for degradation of some organic pollutants under visible light at ambient temperature due to the surface plasmon resonance effect of the noble metal nanoparticles (Ag, Au, Pd) embedded in polymeric films together with ZnO NPs [13,45,46]. In the literature, hybrid composites bearing ZnO and noble metal NPs are reported, with good results for photocatalytic degradation of organic pollutants under visible light [45,46,47]. However, up to now, there are only few studies concerning the use of ZnO and noble metal NPs/polymer systems in photocatalysis, especially in visible domain [13,48], despite the great advantages of these catalytic materials such as easy recovery and reusability. First, the photocatalytic performance of our composite films (M1, M1-Ag, M1-Au and M1-Pd) was evaluated in the decomposition of an organic dye, namely methyl orange (MO), under visible light irradiation. The MO aqueous solution (5 × 10^−5^ M) subjected to the above conditions was analysed through UV-vis spectroscopy, technique appropriate for the nanocatalysis field to monitor the reaction kinetics [49]. A blank experiment was also realized, the pure MO solution in the absence of the photocatalyst was subjected to visible light irradiation for 300 min, the degradation degree attained being 6.2% (Table 2), which was assigned to MO photolysis, not decomposition.

The evolution of MO concentration as a function of the irradiation time, in the presence of our hybrid films, was evaluated with the ratio A_t_/A_0_ = C_t_/C_0_ (A_t_—absorbance at any time “t,” A_0_—absorbance at t = 0, C_t_—MO concentration at any time “t,” C_0_ is the MO initial concentration) and the photodegradation degree (D) was estimated with the Equation (3):

D = (1 − C_t_/C_0_) × 100 = (1 − A_t_/A_0_) × 100. (3)

The photodegradation of MO in the presence of our catalysts (M1, M1-Ag, M1-Au and M1-Pd) exhibited pseudo first order kinetics and were investigated by correlating the ln(C_0_/C_t_) and the reaction time, the slope of the straight fitting line of this graphical representation giving the rate constants (k) of the photoreactions.

**Figure 9:**
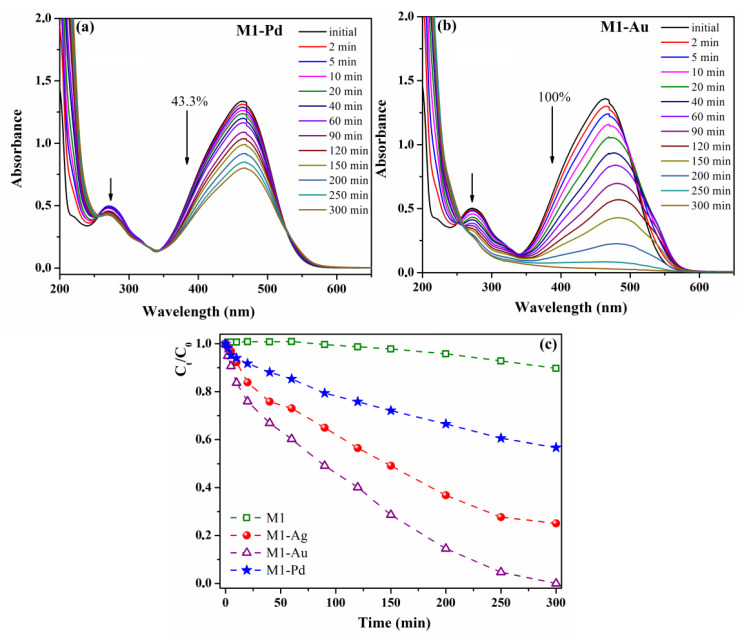
Modification of UV-vis absorption spectrum of methyl orange aqueous solution during visible light irradiation in the presence of M1-Pd film (**a**) or M1-Au film (**b**); temporal evolution of methyl orange photodegradation efficiencies (C_t_/C_0_) in the presence of hybrid films containing modified ZnO NPs (M1, M1-Ag, M1-Au, M1-Pd) (**c**).

As we anticipated, M1 composite film containing only Sil-ZnO NPs (active only in UV light, E_g_ = 3.133 eV) did not show photocatalytic activity under visible light irradiation. The MO degradation degree in the presence of M1 after 300 min of visible irradiation is only 10.3% (k = 0.26 × 10^−3^ min^−1^, Table 2), mainly due to MO photolysis and adsorption over the polymeric material. These results together with those from blank experiment indicate that the adsorption of methyl orange over the photocatalytic material and MO photolysis are both minor and further can be neglected. The supplementary addition of various noble metals nanoparticles (Ag, Au or Pd) to the M1 film led to photocatalytic materials (M1-Ag, M1-Au or M1-Pd) active in visible light, with good efficiency in degrading pollutants from water. Figure 9a,b show the improvement attained in MO degradation in the presence of M1-Pd/Au due to the surface plasmon resonance effect.

The intensity of the methyl orange characteristic absorption bands at about 465 nm and 273 nm significantly decrease with increasing the visible light irradiation time. The photocatalytic efficiency of our composite films is discriminated by the noble metal used in their composition, as can be seen in Figure 9c. Thus, the performance of the investigated catalysts on the MO degradation parameters (degradation degree and degradation rate) after 300 min of exposure to visible radiation varies as follows—M1-Pd (43.3%, k = 2.04 × 10^−3^ min^−1^) < M1-Ag (74.9%, k = 4.87 × 10^−3^ min^−1^) < M1-Au (100%, k = 10.22 × 10^−3^ min^−1^). The noble metal nanoparticles photogenerated in the polymer matrix (Au, Ag or Pd) have almost the same dimensions (~5–10 nm) and it is well-known that the catalytic performance depends on the surface area, which increases with decreasing NPs sizes [37,50,51]. However, it has been observed that they induce a different photocatalytic efficiency of the final materials under the action of visible light. This finding can be attributed to a different dispersion of nanoparticles in the organic component and as can be observed from the TEM images, Pd NPs have a higher tendency to agglomerate, while Au NPs are uniformly distributed in the matrix.

The agglomeration tendency of nanoparticles leads to a decrease of their active surface area and implicitly to the reduction of their photocatalytic activity and therefore the M1-Pd composite showed the lowest efficiency in MO photodegradation. Also, the photogeneration of Au NPs mainly around ZnO NPs (as was observed from TEM) is another factor that contributes to an improved photocatalytic activity of M1-Au film. Another plausible explanation for the better photocatalytic efficiency of M1-Au film is that Au NPs have a *4f* electronic configuration and the pollutant molecules form complexes more easily by the interaction of functional groups of organic derivatives with the f-orbital than with d-orbital of Ag/Pd NPs [52].

The photocatalytic mechanism of the composites containing Sil-ZnO and noble metal NPs is based on the equilibration of the Fermi levels of noble metal (Ag, Au or Pd) and ZnO to the same value through the transfer of electrons from Fermi level of noble metal to Fermi level of ZnO and so the composite material have more free electrons above the new Fermi level [43,53]. Since, in our case, Au NPs are in the neighbourhood of ZnO NPs, it is easier to reach this equilibrium than for the composites containing Ag or Pd NPs and in this condition, the M1-Au composite has a better photocatalytic activity than M1-Ag and M1-Pd. Furthermore, the electrons from equilibrium Fermi level under visible light irradiation are excited to ZnO conduction band due to surface plasmon resonance effect of the noble metal nanoparticles. The excited electrons interact with oxygen molecules generating highly reactive oxygen radical species (•O^2−^), which further react with H_2_O producing hydroxyl radicals (OH•). At this point, the free radicals OH• and •O^2−^ cause the degradation of organic compounds into CO_2_ and H_2_O.

**Figure 10:**
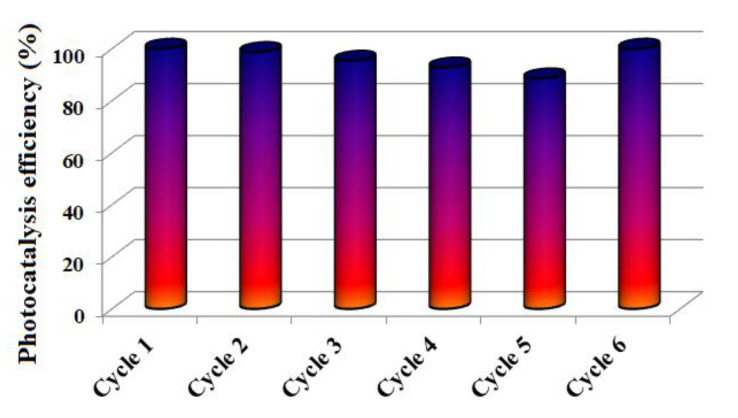
Evolution of the photocatalytic efficiency of M1-Au hybrid film in the degradation of methyl orange after 6 cycles under visible light irradiation (300 min). Cycle 6: after 5 successive cycles, M1-Au film is left 24 h in water and 24 h under normal environmental conditions and after that a new cycle is performed.

The photocatalyst reusability is a very important aspect for practical applications and therefore, in the next experiments, the M1-Au composite was used for methyl-orange photodegradation for six cycles and the obtained results are presented in Figure 10.

The test was done as follows—the MO degradation was followed in the presence of M1-Au catalyst for five successive cycles and it was observed that its efficiency decreases slightly, from 100% to ~89%. After these repetitive cycles, the M1-Au film was kept in water for 24 h and then another 24 h under normal environmental conditions and used again in cycle 6 in the degradation of the methyl-orange dye in aqueous solution under the action of visible light (300 min). It was observed that the photodegradation efficiency returned to the initial value of 100%. As a short conclusion, our hybrid films with ZnO nanoparticles and noble metals can be reused multiple times and if the photocatalytic efficiency decreases, their regeneration is realized simply by keeping them in water and then in environmental conditions.

**Figure 11:**
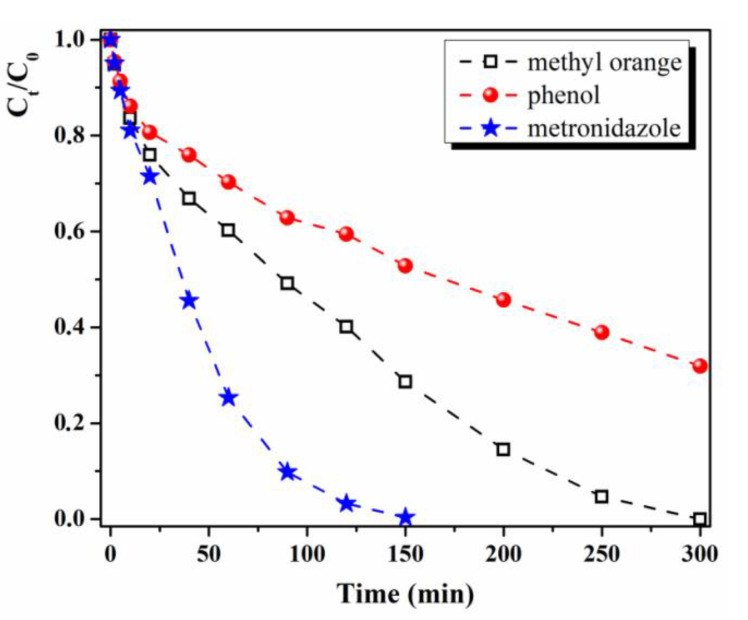
Temporal evolution of reduction efficiencies (C_t_/C_0_) of methyl-orange, phenol and metronidazole in the presence of M1-Au film under visible irradiation.

Additionally, the M1-Au hybrid composite was tested as photocatalyst (in visible light) for the degradation of other types of pollutants which are often found in wastewater, namely phenolic derivatives (phenol) and pharmaceutical compounds (metronidazole). Very good results were obtained in the decomposition of metronidazole, which is completely photodegraded after only 150 min of exposure to visible light in the presence of M1-Au hybrid material (Figure 11), with a constant rate k = 31.53 × 10^−3^ min^−1^.

The results are promising for such a material in which the catalyst nanoparticles have been immobilized in a polymer matrix, as similar results for metronidazole degradation achieved only in the presence of catalyst particles are presented in the literature. For example, TiO_2_ NPs photodegrade 97.6% metronidazole after 180 min of UV irradiation (k = 5.13 × 10^−2^ min^−1^) [54] and ZnO-ZnAl_2_O_4_ catalyst induce only 90% metronidazole photodegradation after 300 min of exposure to solar light (k = 5.13 × 10^−2^ min^−1^) [55]. The photocatalytic efficiency of TiO_2_ polymeric composites (chitosan-TiO_2_ or polyvinyl alcohol-chitosan-TiO_2_) under UV light in metronidazole degradation is 100% after 120 min of reaction time (k ~4.1–4.6 × 10^−2^ min^−1^) [56].

As can be observed from Figure 11, in the case of phenol photodegradation, the M1-Au film had weaker catalytic activity, the photodegradation degree attained after 300 min of visible light exposure being around 68% (k = 4.0 × 10^−3^ min^−1^).

The metronidazole photocatalytic degradation take place easier since its decomposition pathway is simpler and involves the formation of fewer intermediates—oxidation of the ethanol group followed by decarboxylation and carbon oxidation stage yielding to CO_2_ and H_2_O [57]. The methyl orange photodegradation implies first demethylation of the compound, then azo bond (–N=N–) breaking take place with the formation of new intermediates (N,N-dimethylbenzenamine, N,N-dimethyl-p-phenylenediamine and sulfanilic acid), which further are decomposed into sulfanilic acid and other small molecule organics or even into carbon dioxide and water [58,59]. In the case of photocatalytic decomposition of phenol, in the first stage take place the formation of new products (hydroquinone, pyrocatechol, 1,2,4-benzenetriol, pyrogallol, 2-hydroxy-1,4-benzoquinone, 1,4-benzoquinone) by the reaction of phenol with·OH radical ions, step that prolongs the reaction time for phenol photodecomposition. The new products undergo further photocatalytic oxidation and produce very polar intermediates like carboxylic acids and aldehydes and finally CO_2_ and H_2_O [60]. So, our hybrid materials comprising ZnO NPs and noble metal nanoparticles (especially Au NPs) are recommended for removing dyes (methyl orange) and pharmaceutical products (metronidazole) from waste waters from various industries.

## 4. Conclusions

Flexible self-standing nanocomposite films bearing silane-functionalized ZnO nanoparticles and in situ photogenerated noble metal nanoparticles (Ag, Au or Pd) were easily synthesized by the simple one-pot photopolymerization of urethane dimethacrylate monomers derived from bio-based castor oil and polypropylene diol. The films with both ZnO and noble metal NPs can be used as active photocatalysts under visible light with an appropriate efficiency in dyes degradation (methyl orange). The Sil-ZnO/Au catalyst displayed an improved photocatalytic activity (degradation degree of MO is 100% after 300 min) comparative with Sil-ZnO/Ag and Sil-ZnO/Pd hybrid materials, behaviour mainly attributed to the formation of Au NPs in the vicinity of ZnO NPs or even in contact with them which causes a synergistic effect that determine the augmentation of catalytic competence. Sil-ZnO/Au nanocomposite is also a good catalyst for phenol photodecomposition (68% after 300 min) but the best result were obtained for metronidazole degradation (100% after 150 min). The hybrid film can be reused for multiple runs without major loss of photocatalytic efficiency and its regeneration can be achieved very simply by keeping the film in water and then in environmental conditions.

## Figures and Tables

**Table 1:** Gravimetric composition of the investigated hybrid materials (each formulation contains 1 wt.% Irgacure 819).

Sample	CO-UDMA-Si	PPO-UDMA	Sil-ZnO	AgNO_3_	HAuCl_4_·3H_2_O	Pd(NO_3_)_2_·2H_2_O
M0	70	30	0	0	0	0
M1	70	30	1	0	0	0
M1-Ag	70	30	1	1.6	0	0
M1-Au	70	30	1	0	2.0	0
M1-Pd	70	30	1	0	0	2.5

**Table 2:** Photodegradation parameters (degradation degree and apparent rate constant, k) for the decomposition of different pollutants (methyl orange, phenol, metronidazole) under visible light irradiation in the presence of polymeric composite films M1, M1-Ag, M1-Au or M1-Pd.

Pollutant	Catalyst	Time (min)	Degradation Degree (%)	k (min^−1^)	Regression Coefficient (R^2^)
Methyl orange	No catalyst	300	6.2	0.15 × 10^−3^	0.857
	M1	300	10.3	0.26 × 10^−3^	0.811
	M1-Ag	300	74.9	4.87 × 10^−3^	0.995
	M1-Au	300	100	10.22 × 10^−3^	0.966
	M1-Pd	300	43.3	2.04 × 10^−3^	0.985
Phenol	No catalyst	300	7.1	0.35 × 10^−3^	0.978
	M1-Au	300	68.1	4.00 × 10^−3^	0.977
Metronidazole	No catalyst	150	9.8	0.49 × 10^−3^	0.934
	M1-Au	150	99.7	31.53 × 10^−3^	0.959
